# Drought Stress Induced Different Response Mechanisms in Three Dendrobium Species under Different Photosynthetic Pathways

**DOI:** 10.3390/ijms25052731

**Published:** 2024-02-27

**Authors:** Ke Xia, Qiaofen Wu, Yanni Yang, Qiao Liu, Zaihua Wang, Zhiguo Zhao, Jie Li, Jinxiang He, Shengfeng Chai, Shuo Qiu

**Affiliations:** 1 Guangxi Institute of Botany, Guangxi Zhuang Autonomous Region and Chinese Academy of Sciences/Guangxi Key Laboratory of Plant Functional Phytochemicals and Sustainable Utilization, Guilin 541006, China; xiake4502@163.com (K.X.); wuqfmj@163.com (Q.W.); yangyanni219@126.com (Y.Y.); gs.liuqiao20@gzu.edu.cn (Q.L.); 13788588632@139.com (Z.Z.); hjxiang202@163.com (J.H.); sfchai@163.com (S.C.); 2 Guangdong Provincial Key Laboratory of Ornamental Plant Germplasm Innovation and Utilization, Environmental Horticulture Research Institute, Guangdong Academy of Agricultural Sciences, Guangzhou 510640, China; wangzaihua@163.com (Z.W.); goodlijie666@163.com (J.L.)

**Keywords:** carbon metabolism, drought tolerant, phosphoenolpyruvate carboxylase, enrichment analysis, *Dendrobium*

## Abstract

Many *Dendrobium* species, which hold a high status and value in traditional Chinese medicine, grow on barks and rocks in the wild, often encountering harsh environments and facing droughts. However, the molecular mechanisms underlying the shift in the photosynthetic pathway induced by drought remain unclear. To address this issue, three *Dendrobium* species with different photosynthetic pathways were selected for sequencing and transcriptome data analysis after drought treatment. The findings included 134.43 GB of sequencing data, with numerous Differentially Expressed Genes (DEGs) exhibiting different response mechanisms under drought stress. Gene Ontology (GO)–KEGG-based enrichment analysis of DEGs revealed that metabolic pathways contributed to drought tolerance and alterations in photosynthetic pathways. Phosphoenolpyruvate Carboxylase (PEPC) was subjected to phylogenetic tree construction, sequence alignment, and domain analysis. Under drought stress, variations were observed in the PEPC gene structure and expression among different *Dendrobium* species; the upregulation of *Dc_gene2609* expression may be caused by dof-miR-384, which resulted in the shift from C_3_ photosynthesis to CAM, thereby improving drought tolerance in *Dendrobium*. This study revealed the expression patterns and roles of PEPC genes in enhancing plant drought tolerance and will provide an important basis for in-depth research on *Dendrobium*’s adaptation mechanisms in arid environments.

## 1. Introduction

Drought-induced stress takes precedence among environmental stresses worldwide, markedly affecting agricultural productivity [1]. According to statistics, more than 30% of the world’s land is arid or semi-arid [2]. As the most damaging abiotic stress in agriculture, drought contributes to more yield losses than any other stress factor [3]. Mild drought can affect plant growth and development, while severe drought can directly lead to plant death [4]. Drought stress severely limits plant growth, thereby reducing crop yield and quality [5,6]. The reduction in cyclin-dependent kinase activity during drought leads to slower cell division, causing a marked decline in plant growth rate under water scarcity conditions [7]. Furthermore, drought typically induces a decrease in leaf area, leaf extension rate, and overall leaf quantity. Leaves, under drought stress, commonly exhibit varying degrees of wilting and yellowing [8]. In response to drought, mature plants can mitigate water loss due to leaf transpiration through the closure of leaf stomata [9]. However, stomatal closure also hinders plant photosynthesis [10]. Under drought stress, plant cytoplasm contracts due to dehydration, resulting in mechanical damage to the cell membrane. After cytoplasmic dehydration, the concentration of intracellular solutes increases, increasing the possibility of protein–protein interactions and, in severe cases, leading to the aggregation and denaturation of intracellular proteins [11]. Additionally, the accumulation of reactive oxygen species in plants under drought stress can cause membrane lipid peroxidation. Malondialdehyde, the final product of plasma membrane peroxidation, can also cause protein inactivation on cell membranes, thereby compromising the structure and function of biofilms [7].

In response to prolonged water deficit stress, plants have evolved coping mechanisms to increase their drought tolerance through physical adaptations, molecular regulations, and environmentally suitable metabolic pathways [12,13,14,15]. For example, some *Dendrobiums* can resist drought by using the photosynthetic pathway of crassulacean acid metabolism (CAM) for efficient water utilization [16,17].

*Dendrobium catenatum*, an epiphytic orchid belonging to the *Dendrobium* genus (Orchidaceae), is mainly distributed in Asia, with widespread cultivation and use in China, growing primarily on barks and rocks in nature [18,19]. Traditional Chinese Medicine (TCM) dates back thousands of years, and *D. catenatum* is regarded as the first of the nine great immortal herbs for its high medicinal value [20,21]. Many *Dendrobium* species, including *D. nobile*, *D. fimbriatum*, *D. loddigesii*, and *D. catenatum*, have long been used as a TCM to treat chronic diseases, making a significant contribution to public health and promoting TCM development, as it has antibacterial and antiaging properties [22,23,24]. As of 2022, the industrial cultivation of *D. catenatum* in China surpassed 30,000 hectares, contributing to a comprehensive output value exceeding CNY 50 billion [21].

Prior to artificial cultivation, the harsh wild environment allowed *Dendrobium* to evolve varying mechanisms to withstand stresses such as drought. *Dendrobium* has evolved succulent storage organs such as pseudobulbs and thick leaves, which facilitate drought tolerance in terms of the plant’s histological morphology [25,26,27]. By testing the physiological indicators and secondary metabolites of *D. catenatum*, Wu et al. [8] found that increased antioxidant enzyme activity and osmotic pressure markedly contribute to the plant’s response to drought stress. Comparing the two published genomes from epiphytic orchid species (*Phalaenopsis equestris* and *D. catenatum*) *D. catenatum* possessed more heat shock 70 kDa protein family members and R genes, suggesting that *D. catenatum* can tolerate diverse environments and has superior qualities to tolerate adverse events [28,29]. Overall, studies exploring drought tolerance genes in *Dendrobium* are limited.

The CAM pathway confers drought tolerance to many plants. In *Dendrobium*, different *Dendrobium* species have varying photosynthetic pathways. For example, *D. fimbriatum* is a C_3_ plant that utilizes the Calvin cycle and uses only the C_3_ pathway for photosynthesis. However, some *Dendrobium* species, such as *D. catenatum*, can change between the C_3_ and CAM pathways in response to stress or environmental changes [30]. *D. loddigesii* is a dedicated CAM pathway plant, which means that photosynthesis occurs via the CAM pathway regardless of drought [31,32]. Notably, Phosphoenolpyruvate Carboxylase (PEPC) catalyzing irreversible carboxylation of phosphoenolpyruvate and converting it to oxaloacetate plays an important role in the carbon–nitrogen metabolism of C_3_ and CAM plants [33,34]. As decreased PEPC activities during drought stress reduces the photosynthetic and electron transport rates [35], PEPC may play an important role in plant resistance to drought by regulating photosynthesis.

Genome sequencing studies of *D. catenatum* can provide a good histological basis for studying gene function in this species [29,36,37]. Current research on drought-tolerant *Dendrobium* has focused mainly on genes related to polysaccharide synthesis and degradation [8,29,38]. Related omics studies, as well as other drought-tolerance pathways, remain unclear, especially for different *Dendrobium* species, and no relevant comparative studies are available, highlighting the need for exploring the effects and roles of different photosynthetic metabolic pathways in relation to drought stress. Notably, in *Dendrobium*, the behavior of PEPC genes under drought stress, as well as the molecular mechanisms underlying the shift in the photosynthetic pathway induced by drought, remain unclear. Here, we proposed a hypotheses that there were different underlying molecular mechanisms among different species with different photosynthetic pathway. But how many DEGs exhibited different response and which PEPC gene led to the shift from C_3_ photosynthesis to CAM induced by drought stress in *Dendrobium*? In this study, transcriptome sequencing and analysis were conducted using three *Dendrobium* species with distinct photosynthetic characteristics following drought treatment. The findings from this research will provide an important basis for in-depth research on *Dendrobium*’s adaptation mechanisms in arid environments and provide theoretical support for the selection and breeding of drought-tolerant traits in *Dendrobium*.

## 2. Results

### 2.1. RNA-Seq Analysis under Drought Stress

In this study, three independent sequencing sessions were conducted for each genotype/treatment group, resulting in 18 constructed cDNA libraries. The sample information was showed in Table 1. The raw RNA-seq data generated from this study has been submitted to the Sequence Read Archive of the National Center for Biotechnology Information (NCBI) under accession number PRJNA1055284. In the cDNA libraries of *D. catenatum*, *D. loddigesii*, and *D. fimbriatum*, after removing adaptors, low-quality reads, and low-quality regions, the numbers of clean reads were 43.65 Gb, 45.02 Gb, and 45.76 Gb, respectively. The average clean data of each sample were 6.87 Gb, 7.14 Gb, and 7.39 Gb, respectively, which were used for further analysis. Q30 represents the percentage of bases with an accuracy above 99.9% and was >92.34%, >92.40%, and >92.03% for the three *Dendrobium* species, respectively. Sequence alignment was performed between the clean reads of various samples of *D. catenatum* and the designated reference genome [29], with alignment efficiencies ranging from 83.72% to 85.31%. Table 2 contains the statistical data on the output of the sequencing data for each sample. Based on the comparison results, variable splicing prediction analysis, gene structure optimization analysis, new gene exploration, and gene expression analysis were conducted. DEGs were identified based on their expression levels in different samples, followed by functional annotation and enrichment analysis.

Due to the low efficiency of comparison with the reference genome, the other two *Dendrobium* species were analyzed without a reference genome. Of these, a total of 100,695 unigenes were obtained from the *D. loddigesii* assembly, of which 19,260 unigenes were more than 1 kb in length and the N50 of unigenes was 1389. A total of 56,054 unigenes were obtained from the *D. fimbriatum* assembly, of which 18,953 unigenes were above 1 kb in length and the N50 of unigenes was 1, 858. Both assemblies have high integrity, and Table 3 lists the specific statistical information.

### 2.2. Gene Annotation and DEG Identification

**Figure 1:**
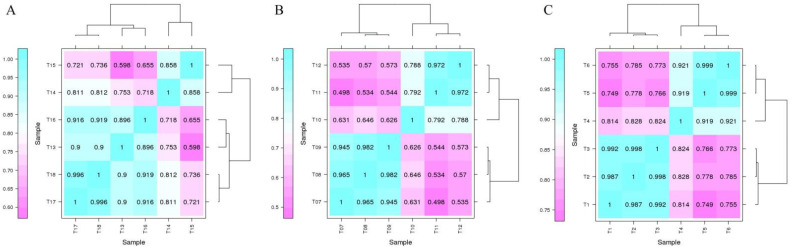
Correlation heatmap and clustering between samples. (**A**): *Dendrobium catenatum*; (**B**): *Dendrobium loddigesii*; (**C**): *Dendrobium fimbriatum*. As the number in the figure tends to 1, the correlation between the two duplicate samples strengthened.

By comparing and analyzing the gene and unigene sequences with databases such as NR, Swiss Prot, GO, COG, KOG, KEGG, and Pfam in *D. catenatum*, 24,876 genes were detected in the drought-treated and control samples, of which 1745 new genes were discovered and 964 new genes were functionally annotated. Annotation results were obtained for 39,254 and 28,208 unigenes in *D. loddigesii* and *D. fimbriatum*, respectively. Subsequently, quantitative analysis was performed on the expression of these sequences. Pearson’s correlation coefficient analysis was conducted based on the expression levels of genes and unigenes in each sample, and the results showed good reproducibility between biological duplicate samples of *D. loddigesii* and *D. fimbriatum*. However, T13 in the control group of *D. catenatum* did not show good repeatability compared to T14 and T15, so T13 was excluded from subsequent analysis (Figure 1) (Supplemental Figure S1).

**Figure 2:**
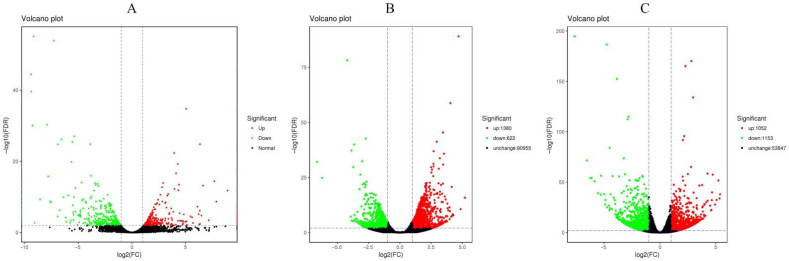
Volcano maps of DEGs in two sets of samples. (**A**): *Dendrobium catenatum*; (**B**): *Dendrobium loddigesii*; (**C**): *Dendrobium fimbriatum*. Each point in the differential expression volcano map represents a gene, and the horizontal axis represents the logarithmic value of the multiple expression differences of a certain gene in the two samples. The vertical axis represents the negative logarithmic value of the statistical significance of changes in gene expression. The larger the absolute value of the abscissa, the greater the difference in expression multiples between the two samples. The larger the ordinate value, the more significant the differential expression, and the more reliable the DEGs screened. The green dots in the figure represent downregulated DEGs, the red dots represent upregulated DEGs, and the black dots represent non-DEGs.

According to the analysis of the expression levels of each sample, 634 genes were identified as DEGs in *D. catenatum* after drought treatment compared to the control group (285 upregulated and 349 downregulated genes). In *D. loddigesii*, 2002 Unigenes were identified as DEG after drought treatment compared to the control (1, 380 upregulated and 622 downregulated unigenes). Similarly, in *D. fimbriatum*, 2, 205 unigenes were identified as DEGs in the drought-treated samples compared to the control (1, 052 upregulated and 1, 153 downregulated unigenes) (Figure 2).

### 2.3. Clustering and Functional Categorization of DEGs

**Figure 3:**
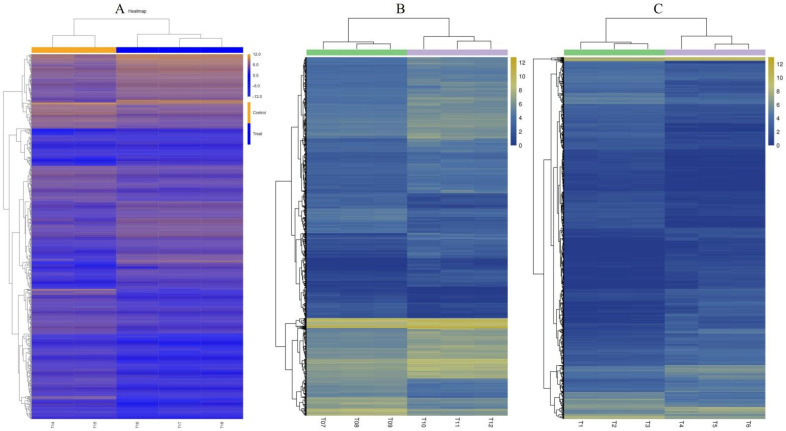
Clustering of DEGs between samples. (**A**): *Dendrobium catenatum*; (**B**): *Dendrobium loddigesii*; (**C**): *Dendrobium fimbriatum*. The horizontal axis represents the sample name and clustering results of the sample, while the vertical axis represents the clustering results of the differential genes and genes. The different columns in the figure represent different samples, and the different rows represent different genes. The color represents the expression level of genes in the sample, and the logarithmic value of FPKM is based on 2.

**Figure 4:**
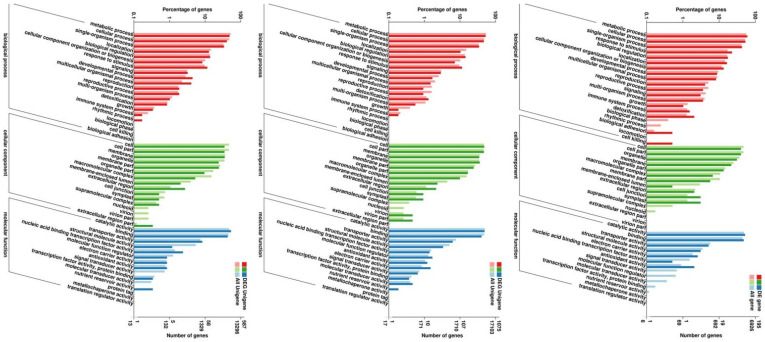
Statistical chart of GO annotation classification for DEGs. The three coordinate maps are *Dendrobium catenatum*, *Dendrobium loddigesii*, and *Dendrobium fimbriatum*, respectively. The horizontal axis represents GO classification, the left side of the vertical axis represents the percentage of genes, and the right side represents the number of genes.

**Figure 5:**
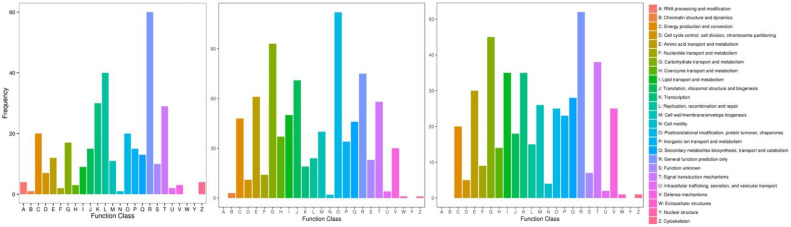
COG annotation classification statistics of DEGs. The three coordinate maps are *Dendrobium catenatum*, *Dendrobium loddigesii*, and *Dendrobium fimbriatum*, respectively. The horizontal axis represents the classification content of COG, and the vertical axis represents the number of genes.

Hierarchical clustering analysis was performed on the DEGs screened by comparison of each treatment, and genes with the same or similar expression patterns were clustered. Figure 3 shows the DEG clustering results. Each set of comparisons had a high number of DEGs, and to further clarify the drought-induced response, we needed to determine in which functional groups and pathways the differential genes were mainly enriched. GO enrichment analysis of thees DEGs was performed. The results showed that both *D. catenatum* or *D. loddigesii* and *D. fimbriatum* were enriched to three functions: biological process, cellular component, and molecular function (Figure 4). Biological processes mainly involve metabolic, cellular, and single-organism processes; cellular components mainly involve cells, cell parts, organelle, and membrane; and molecular functions mainly involve catalytic activity and binding. In the GO functional enrichment analysis, the three *Dendrobium* types showed relatively consistent performance after drought treatment. The DEG analysis combined with the COG (Cluster of Orthologous Groups of proteins) database shows that, after drought treatment, the DEGs in *D. catenatum* were mainly concentrated in general function prediction only, replication, recombination, and repair. For *D. loddigesii*, the DEGs were mainly involved in posttranslational modification, protein turnover, chaperones, carbohydrate transport, and metabolism. In *D. fimbriatum*, functions such as general function prediction only, carbohydrate transport, and metabolism were mainly enriched (Figure 5).

**Figure 6:**
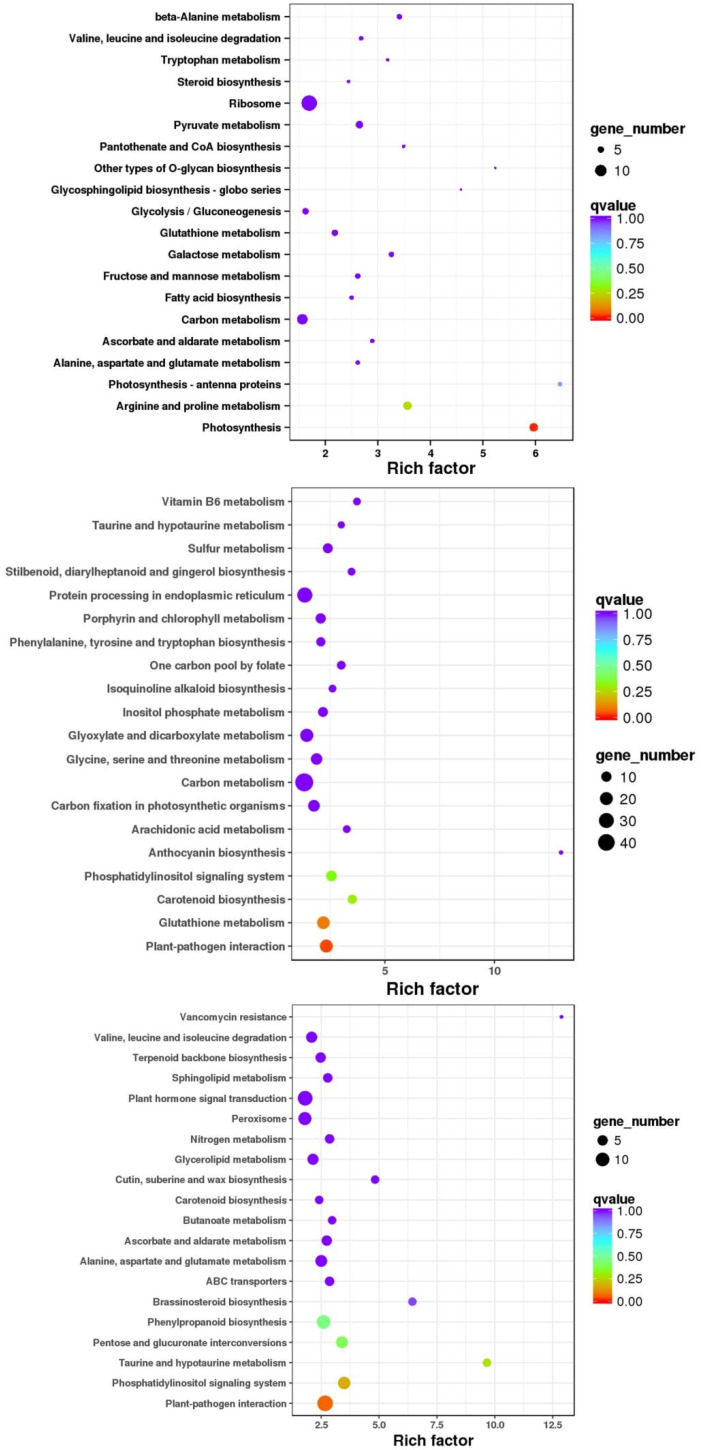
Scatter plot of the KEGG pathway enrichment of DEGs. The three coordinate maps are *Dendrobium catenatum*, *Dendrobium loddigesii*, and *Dendrobium fimbriatum*, respectively. Each circle in the figure represents a KEGG pathway, the vertical axis represents the pathway name, and the horizontal axis represents the enrichment factor, which represents the ratio of the proportion of genes annotated to a certain pathway in differential genes to the proportion of genes annotated to that pathway in all genes. The larger the enrichment factor, the more significant the enrichment level of DEGs in this pathway. The color of the circle represents the q-value, which is the *p*-value corrected for multiple hypothesis tests. The smaller the q-value, the more reliable the enrichment significance of DEGs in this pathway. The size of the circle indicates the number of genes enriched in the pathway, and the larger the circle, the more genes there are.

The annotation results of DEGs classified based on the pathway types in KEGG (Supplemental Figure S2) were as follows: DEGs in *D. catenatum* were mainly concentrated in the ribosome and carbon metabolism pathway; DEGs in *D. loddigesii* were mainly concentrated in the carbon metabolism, ribosome, and biosynthesis of amino acid pathway; and DEGs in *D. fimbriatum* were mainly concentrated in the plant–pathogen interaction, plant hormone signal transduction, carbon metabolism, and phenylpropanoid biosynthesis pathway. Subsequently, the enrichment factor was used to analyze the enrichment degree of the pathway, and Fisher’s exact test method was used to calculate enrichment significance. The top 20 pathways with the most reliable enrichment significance (i.e., the lowest *q*-value) were selected (Figure 6). In *D. catenatum*, the pathways with a higher number of enriched genes were ribosomes, and the pathways with a higher level of enrichment were photosynthesis-antenna proteins. Among them, enrichment significance in the photosynthesis pathway was the most reliable, and the degree of enrichment was highly significant. In *D. loddigesii*, the pathways with a high number of enriched genes were carbon metabolism, and the pathways with a significant level of enrichment were anthocyanin biosynthesis, with the most reliable enrichment significance in the plant–pathogen interaction pathway. Similarly, in *D. fimbriatum*, the pathways with a higher number of enriched genes were plant–pathogen interaction, the pathways with a higher level of enrichment were vancomycin resistance, and the enrichment significance in the plant–pathogen interaction pathway was the most reliable.

### 2.4. Multiple Sequence Alignment of the Three Dendrobium PEPCs

**Figure 7:**
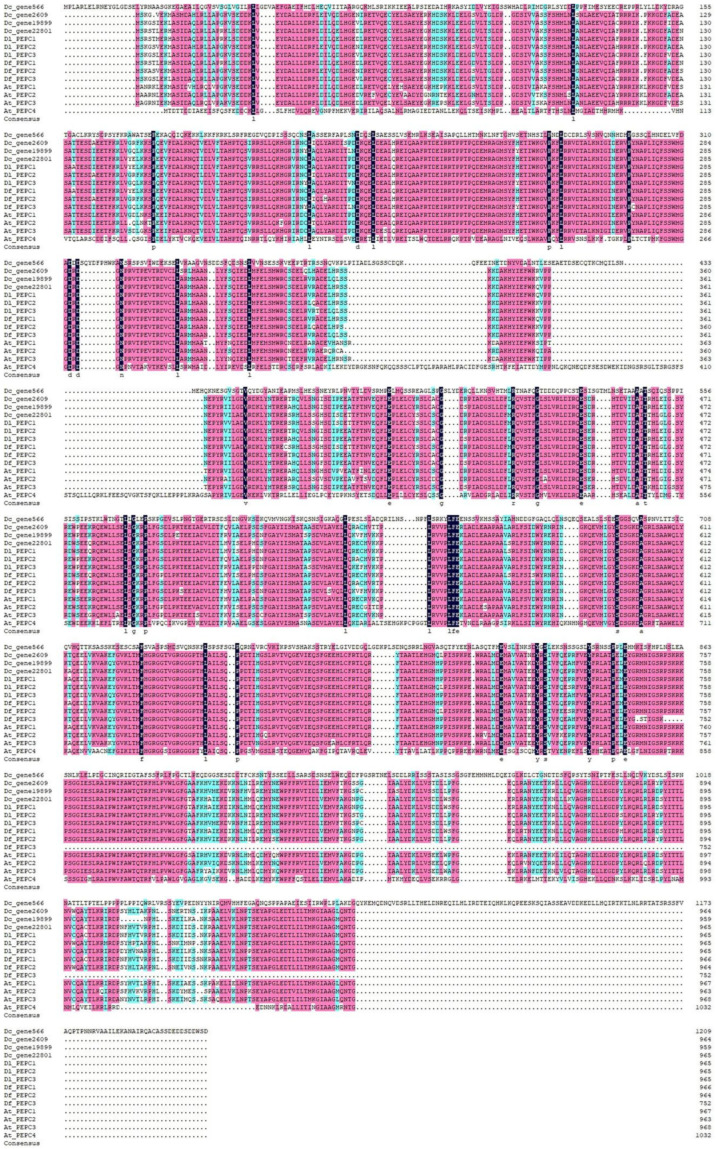
Sequence alignment of the PEPC protein. Different colors represent different sequence similarities. Spots represent gaps.

PEPC gene sequences related to *D. catenatum*, *D. loddigesii*, and *D. fimbriatum* were considered. Because *D. catenatum* had a reference genome, the PEPC gene ID and its sequence were retrieved directly from transcriptome sequencing and annotation files. However, *D. loddigesii* and *D. fimbriatum* lacked a reference genome and thus did not splice the complete gene sequence. In our previous research, we used RACE-PCR technology to obtain the corresponding cDNA sequence. A total of 10 PEPC coding genes were identified. Meanwhile, we utilized the PEPC gene of *Arabidopsis* as a reference for analysis. DNAMAN V7 was used to perform multiple sequence alignments for all sequences directly obtained (see the results in Supplemental Figure S3). The results showed that the PEPC gene, as a whole, had high conservatism. Only the sequences of *Dc_gene566* and *At_PEPC4* exhibited low homology. However, *Dc_gene19899* exhibited a relatively unique performance, with the sequence length markedly exceeding those of other sequences. Concurrently, a significant difference existed between the first half sequence and other sequences, but the second half sequence and other sequences had high alignment efficiency. The sequences obtained by RACE-PCR were amplified to noncoding regions. Through sequence alignment, the positions of the start and stop codons were determined. After removing the noncoding region sequence, we re-aligned the cDNA and translated the protein sequence (see the results in Figure 7; Supplemental Figure S4).

### 2.5. Structure and Phylogenetic Analysis of the PEPC Proteins

**Figure 8:**
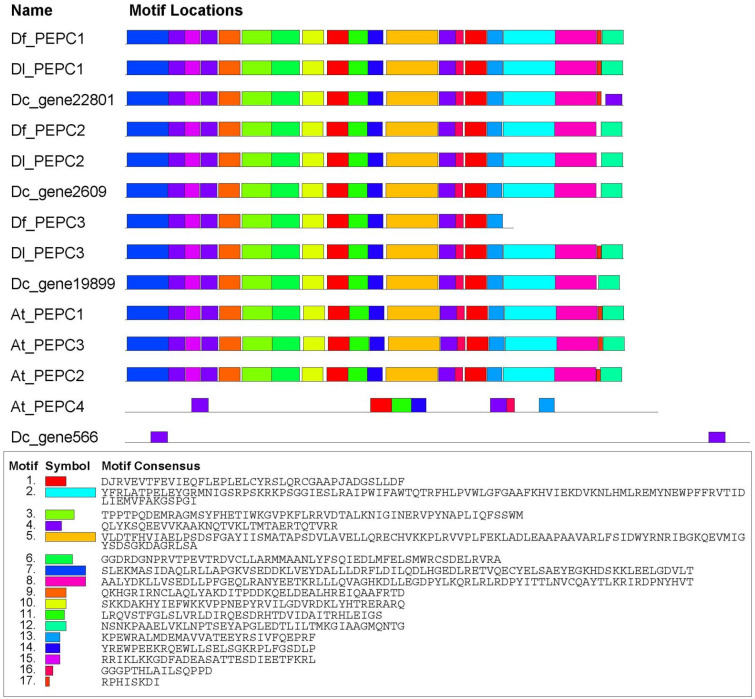
Distribution of conserved motifs in PEPC family members. The color blocks and sequences corresponding to the different motifs are displayed below.

To reveal the structure of the *Dendrobium* PEPC proteins, we used SMART analysis and MEME tools to perform domain and motif analyses on 14 PEPC proteins, including *Arabidopsis*. The prediction of protein domains showed that almost all of these proteins contained conservative PEPCase domains, except *Dc_gene566*, while At_PEPC4 had two PEPCase domains (Supplemental Figure S5). The structure of the *Dc_gene19899* agreed with that of sequence alignment. The second half of the sequence showed the same domain as the other sequences, and the first half of the sequence was predicted to be RPT1. By blasting in the NCBI, the RPT1 sequence was characterized as an uncharacterized protein. The cDNA alignment results showed that the second half of the sequence also had the same start codon as the other sequences. Therefore, the second half of the sequence containing the PEPCase domain was taken as the full length of *Dc_gene19899* in this study. In addition, we obtained the recognition map of the conserved sequence in the protein through the MEME tool, with different colors representing different conserved sequences (Figure 8). Intuitively, the graph shows multiple conserved motifs in the amino acid sequence encoded by the PEPC gene. Slight differences were observed in the number and types of motifs contained in PEPC proteins from different materials, indicating the high degree of conservatism of PEPC family proteins. Similarly, the motifs identified in At_PEPC4 and *Dc_gene566* differed significantly from the other sequences, with only two motif 4 identified in *Dc_gene566*. In addition, due to the earlier termination of Df_PEPC3, motif 2, motif 8, motif 17, and motif 12 were missing. Supplemental Figure S6 shows the 17 identified conserved motif information.

**Figure 9:**
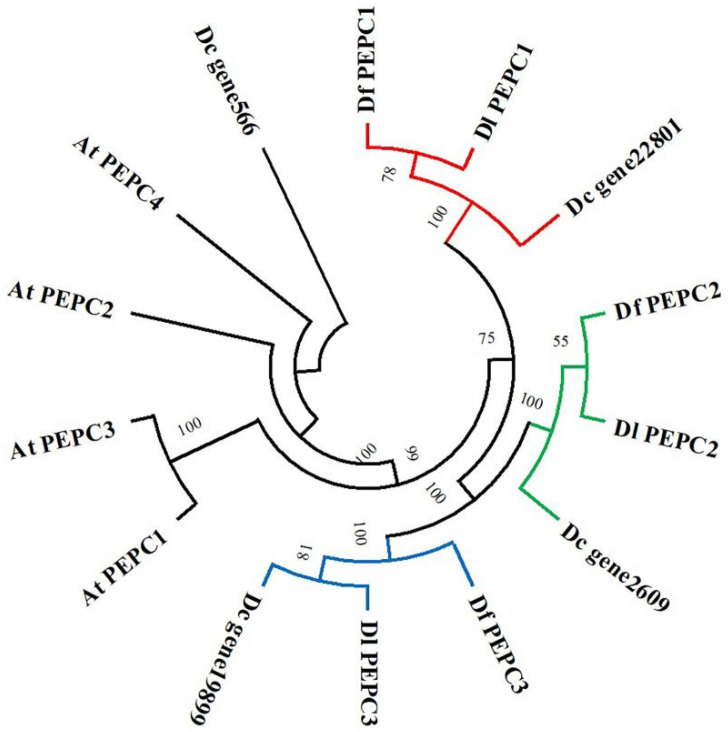
Phylogenetic analysis of *Dendrobium* and *Arabidopsis* PEPC proteins. The full-length amino acid sequences of 14 PEPC proteins were used to construct the neighbor-joining tree using MEGA V11 with 1000 bootstrap replicates. Branches with less than 50% bootstrap support were collapsed. Different branches have different colors.

Here, the neighbor-joining method of MEGA V11 software was used to construct a phylogenetic tree for 10 *Dendrobium* PEPC proteins and 4 *Arabidopsis* PEPC proteins (Figure 9). The branching of the phylogenetic tree showed that *Dc_gene566* had a relatively distant evolutionary relationship with the other sequences. Based on the results of the sequence alignment and protein structure analysis, we do not believe that *Dc_gene566* belongs to the PEPC family, and we removed it in the following analysis. In addition, compared to *Arabidopsis*, the PEPC proteins of the three *Dendrobium* species had closer homology and evolutionary relationships. Each smaller branch contained one of the PEPC proteins of the three *Dendrobium* species, aligning with the expectations of species evolution. Furthermore, the branching relationship and sequence motif composition of phylogenetic trees were consistent; perhaps the differences in PEPC protein motifs among different branches lead to the exercise of different functions during plant growth and development.

### 2.6. Analysis of miRNA Targets in the PEPC Gene of Dendrobium

After prediction, 18 miRNAs were found to block the normal translation process by binding to the complementary sequences of target genes, while another 47 miRNAs were found to affect protein translation by cutting off the mRNA of the target genes (Supplemental Table S1). In the cDNA sequences of all nine *Dendrobium* PEPCs, each gene had numerous miRNA binding sites, such as, *Dc_gene2609* had the highest number (26 sites) and *Df_PEPC1* had the lowest number (16 sites). Notably, 65 miRNAs may participate in the transcriptional regulation of *PEPC*, with each miRNA acting on different target genes. Among them, dof-miR-365 and dof-miR-384 were the most abundant and could bind to 7 PEPC sequences. Through the prediction of the functions of dof-miR-365 and dof-miR-384 and the retrieval of previous studies, we found that miRNAs homologous to dof-miR-365 have been extensively studied in animals but have not yet been found in plants, and dof-miR-384 is a member of the miR166 family and can actively respond to drought stress. For dof-miR-174, dof-miR-175, dof-miR-176, dof-miR-177, dof-miR-178, dof-miR-240, dof-miR-905, and dof-miR-906, which correspond to six target genes, respectively, but no relevant research on these miRNAs in plants has been found.

### 2.7. Validation of the DEGs Expression

**Figure 10:**
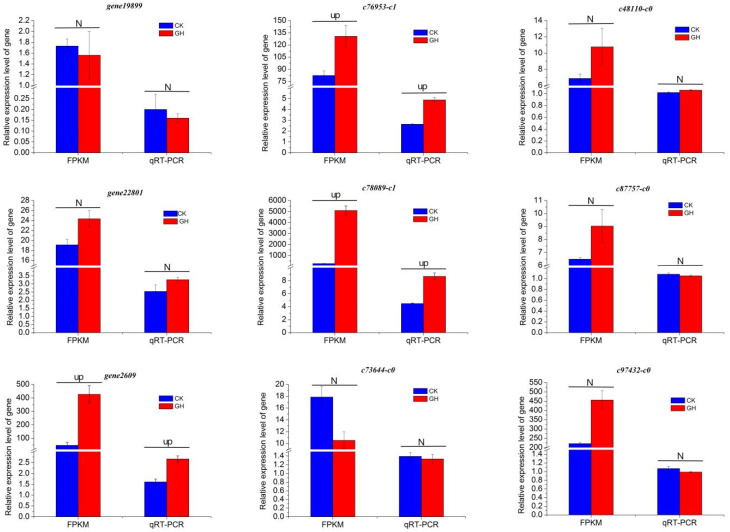
Relative expression levels of nine PEPC genes at transcriptome and mRNA levels. Up indicates that the gene showed up-regulation after drought treatment compared to the control in transcriptome or qRT-PCR expression. N indicates that the gene showed no significant differences after drought treatment compared to the control in transcriptome or qRT-PCR expression.

To validate the transcriptome data, qRT-PCR analysis was conducted using nine PEPC genes associated with the photosynthetic pathway. Supplementary Table S1 provides information regarding these genes and their respective primers. For *c87757-c0* and *c97432-c0* of *D. fimbriatum*, there was a small decline for the qRT-PCR results after drought stress. For the other seven PEPC genes, the qRT-PCR results showed an expression pattern that was consistent with the transcriptome data (Figure 10). Notably, after drought treatment compared to the control, *gene2609* of *D. catenatum* and gene *c76953-c1* and gene *c78089-c1* of *D. loddigesii* showed up-regulation in transcriptome and qRT-PCR expression. However, all genes associated with PEPC in *D. fimbriatum* exhibited no significant differences after drought treatment compared to the control, both in transcriptome and qRT-PCR analyses. These findings suggest that PEPC *gene2609* expression levels may play a crucial role in the regulation mechanism underlying the shift from C_3_ photosynthesis to the CAM pathway in *D. catenatum*.

## 3. Discussion

Orchidaceae is the largest family of flowering plants, and CAM photosynthesis is widespread in this family [39,40]. In *Dendrobium*, obligate CAM, C_3_/CAM intermediate, and obligate C_3_ plant types distributed throughout various habitats [30,31]. Plants that undergo photosynthesis through the CAM pathway close their stomata during the day to reduce water loss and achieve greater drought tolerance [41], highlighting the need to study molecular regulation under drought stress using *Dendrobium* with different photosynthetic types. The widespread use of genome and transcriptome sequencing in plants has unveiled intricate molecular regulations governing life processes, including those in the *Dendrobium* species [42,43]. However, studies on drought stress of *Dendrobium* are few. Wan et al. [44] conducted transcriptome analysis on samples of drought stress of *D. catenatum*. In this study, the transcriptomes of three different types of *Dendrobium* were analyzed, and a more in-depth analysis of differential genes and pathways were compared and analyzed.

In this study, the published genome of *D. catenatum* was used as a reference. Due to the low comparison efficiency of the measurement data for *D. loddigesii* and *D. fimbriatum* relative to *D. catenatum*, a transcriptome without a reference genome was used; this approach was appropriate because the three species selected in this experiment exhibit a relatively distant kinship. As the unigene is a sequence that has not been spliced into a full-length gene, its quantity markedly exceeds the number of genes possessed by *Dendrobium* itself. Similarly, the number of differentially expressed unigenes obtained from transcriptome analysis without reference also markedly exceeded that of *D. catenatum*. Notably, sample T13 in *D. catenatum* had poor repeatability compared to other samples in the same group and was removed, possibly due to errors in our sampling, RNA extraction, or preservation process.

In terms of gene function, differences existed in the predicted results obtained through different databases. In commonly used analyses, such as GO functional enrichment, COG databases, and KEGG databases, the same DEGs may be classified into different categories in different databases. This is related to the classification categories and standards of genes in different databases. Similarly, due to differences in genetic backgrounds and traits among the three *Dendrobium* species, differences existed in the functional types of DEGs produced under the same drought treatment. Although the functional types accounting for a higher proportion of GO functional enrichment results had relatively small differences among the three *Dendrobium* species, careful observation showed differences in the number of DEGs related to the functions of the organelle and membrane. In the C_3_ pathway of *D. fimbriatum*, the number of membranes exceeded that of organelle; in the CAM pathway of *D. loddigesii*, the number of both was basically the same; in *D. catenatum*, the organelle aspect was more numerous than the membrane. Given the distinct effects of C_3_ and CAM pathways on various cellular processes, such as stomatal opening and closing, it is plausible that related DEGs may play a role in the transition from C_3_ to CAM pathways [45]. In addition, DEG classification based on the COG database showed that the functional types of DEGs in the C_3_ pathway of *D. fimbriatum* and the CAM pathway of *D. loddigesii* mainly involve posttranslational modification and protein turnover. Coincidentally, miRNAs can affect mRNA translation, leading to differences in protein levels after translation. Therefore, miRNAs may be involved in drought regulation and the conversion of photosynthetic pathways in *Dendrobium*.

In the prediction of the KEGG database, among the reliable pathways for DEG enrichment in the three *Dendrobium* species, a large proportion belong to photosynthesis, carbon metabolism, and so on. Organic acids includes malic acid are major metabolites in facultative CAM plants [46]. Malic acid can be directly involved in photosynthesis, the citric acid cycle, and other carbon metabolism pathways [47,48]. This is consistent with the results of this study. Under the action of PEPC, malic acid can also affect the movement of stomata, thereby changing the transpiration rate, photosynthesis, and stress resistance of plants [49]. PEPC is a key enzyme that plays a role in photosynthesis and carbon metabolism [50,51]. It can play an important role in plant responses to drought stress and changes in photosynthetic pathways [52,53]. Therefore, an analysis of the PEPC family of three *Dendrobium* species is warranted.

A comparison of the cDNA and protein sequences of PEPC revealed that *Dc_gene566* and *Dc_gene19899* had significant differences compared to other sequences, resulting in differences in protein domains and motifs. As the first half of *Dc_gene19899* lacked any sequence of the PEPC domain, we speculated that this was due to incorrect merging and prediction of the reference genome. Due to the poor conservation of motif 4 in *Dc_gene566* (Supplementary Figure S6), we do not believe that motif 4 is an essential functional region sequence for the PEPC protein. The identified that motifs 1, 11, 14, 16, and 13 were present in all other sequences and exhibited high conservatism; these regions are essential functional region sequence for the PEPC protein. In addition, Df_PEPC3 lacked four motifs compared to the other sequences, which may indicate functional differences between Df_PEPC3 and other PEPC members and require further experimental verification.

Regarding miRNA, current research suggests that 60% of all targets were mapped to the coding sequence [54]. Therefore, our experiment used the coding region sequence to predict miRNA target sites, and we discovered that dof-miR-384 belongs to the miR166 family. The expression level of miR166 is downregulated under drought stress [55]. In this experiment, dof-miR-384 could bind seven PEPC genes, including *Dc_gene2609*, which exclusively showed a significant increase in expression after drought stress. Because miRNA often inhibits the expression of target genes in the cytoplasm, we speculate that dof-miR-384 has a negative regulatory effect on drought tolerance in *D. catenatum*. Compared with the other PEPC genes reported, *Dc_gene2609* belongs to CAM PEPC isoforms [56,57]. Under drought stress, the downregulation of dof-miR-384 expression induced an upregulation of *Dc_gene2609* expression, which subsequently led to stomatal closure and the conversion of the photosynthetic pathway from C_3_ to CAM, thereby improving the drought tolerance of *Dendrobium*. Notably, the function of *Dc_gene2609* and the interaction between dof-miR-384 and *Dc_gene2609* require further experimental verification. After drought treatment compared to the control, all genes associated with PEPC in *D. fimbriatum* exhibited normal expression, but in *D. loddigesii*, *c76953-c1* and *c78089-c1* showed up-regulation expression. Compared with the other PEPC genes reported [56,57], *c76953-c1* and *c78089-c1* belongs to C_3_ *PEPC isoforms* and CAM *PEPC isoforms*, respectively. In future, the functional analysis of *Dc_gene2609*, *c76953-c1*, and *c78089-c1* will be further conducted.

## 4. Materials and Methods

### 4.1. Plant Material and Treatments

Three *Dendrobium* species were selected in this study: *D. fimbriatum* was selected as an obligate C_3_ plant, *D. loddigesii* as an obligate (or constitutive) CAM plant, and *D. catenatum* as a C_3_/CAM intermediate (inducible or facultative CAM) plant [30,31]. All the three wild species were obtained from the laboratory of Professor Shuo Qiu of the Guangxi Institute of Botany, complying with Chinese legislation, and came from Baise City, Guangxi Province, China.

Three *Dendrobium* species were grown and maintained in plastic sheds at the Guangxi Institute of Botany (110°17′ E, 25°01′ N), Guilin, China. The potting medium used was moss and coconut blocks at a ratio of 3:1 (*v*/*v*); the coconut block had a 1.2-cm diameter. All plants were watered with 1/2 HS nutrient solution [58] once a week from March to October during the growing season. From September to November, well-developed plants possessing more than four pairs of leaves were selected and assigned to Well-Watered (WW) and Drought-Stressed (DS) treatments. The WW plants were placed in a climate chamber with light intensity at the level of leaves kept at approximately 250–350 μmol m^−2^ s^−1^ PPFD, a 55%/75% (day/night) of relative air humidity, a 12/12 h (light/dark) photoperiod, and a temperature regime of 28 °C/18 °C (day/night) for at least two weeks. The plants were watered every other day. The climate chamber had a 400 L volume. These conditions satisfied the requirements of the C_3_/CAM intermediate plants to switch to the C_3_ photosynthesis mode. However, the DS plants were placed into other climate chambers with light at approximately 250–350 μmol (photon) m^−2^ s^−1^, 55%/75% (day/night) humidity, and a 12/12 h (light/dark) photoperiod. The plants were kept without watering for at least 21 d to induce the CAM photosynthesis mode in the C_3_/CAM intermediate plants. The middle leaves of the pseudobulbs were selected for measurement after full expansion. Sampling was conducted on treatment day 0 (CK) and treatment day 21. All experiments were conducted in triplicate.

### 4.2. RNA Extraction, Library Construction, and Sequencing

RNA was extracted using an RNAprep Pure Plant Plus Kit (TIANGEN, Beijing, China) and its concentration measured with NanoDrop 2000 (Thermo, Waltham, MA, USA). RNA integrity was assessed using the RNA Nano 6000 Assay Kit of the Agilent Bioanalyzer 2100 system (Agilent Technologies, Santa Clara, CA, USA). A total amount of 1 μg RNA per sample was used as the input material for the RNA sample preparations. Sequencing libraries were generated using the NEBNext UltraTM RNA Library Prep Kit for Illumina (NEB, Ipswich, MA, USA), following the manufacturer’s recommendations. Briefly, the mRNA was enriched using oligo (dT) magnetic beads, followed by RNA fragmentation into short fragments. Using RNA as a template, the first strand cDNA was synthesized with six-base random primers and reverse transcriptase. Second-strand cDNA synthesis was subsequently performed using DNA Polymerase I and RNase H. The resulting double-stranded cDNA was purified with magnetic beads. Ends were then repaired with an adenine tail added to the 3′ tail. In order to select cDNA fragments that were preferentially 240 bp in length, the library fragments were purified with the AMPure XP system (Beckman Coulter, Beverly, MA, USA). Sequencing adaptors were added to the fragments, which were enriched by PCR amplification. Finally, PCR products were purified (AMPure XP system), and library quality was assessed on the Agilent Bioanalyzer 2100 system. The library preparations were sequenced on an Illumina platform, and paired-end reads were generated.

### 4.3. Quality Control and Transcriptome Assembly

Raw data (raw reads) of the fastq format were first processed through in-house perl scripts. In this step, clean data (clean reads) were obtained by removing reads containing adapter, reads containing ploy-N, and low-quality reads from the raw data. Q20, Q30, GC content, and sequence duplication level of the clean data were also calculated. All the downstream analyses were based on clean high-quality data. The adaptor sequences and low-quality sequence reads were removed from the datasets. Raw sequences were transformed into clean reads after data processing and then mapped to the reference genome sequence. Only reads with a perfect match or one mismatch were further analyzed and annotated based on the reference genome. Hisat2 tools soft were used to map the reference genome. For plants without a reference genome, another method was used to assemble the transcripts. The left files from all libraries/samples were pooled into one large left.fq file, and the right files into one large right.fq file. Transcriptome assembly was accomplished based on the left.fq and right.fq using Trinity [59] with min_kmer_cov set to 2 by default and all other parameters set to default.

### 4.4. Quantification of Gene Expression and Differential Expression Analysis

Quantification of gene expression levels: Gene expression levels were estimated by fragments per kilobase of transcript per million fragments mapped. The read count value for each gene was assumed to be the original expression amount of a gene using HTSeq statistics [60]. The read count was positively correlated with the true gene expression level, gene length, and sequencing depth. Differential expression analysis of the two conditions/groups was performed using the DESeq R package (1.10.1). DESeq provides statistical routines for determining differential expression in digital gene expression data using a model based on a negative binomial distribution. The resulting *p*-values were adjusted using Benjamini and Hochberg’s approach for controlling the false discovery rate. Genes with an adjusted *p* < 0.05 found by DESeq were assigned as differentially expressed.

### 4.5. Analyses of GO and KEGG Pathways

Gene function was annotated based on the following databases: NCBI non-redundant protein sequences (Nr); NCBI non-redundant nucleotide sequences (Nt); Protein family (Pfam); Clusters of Orthologous Groups of proteins (KOG/COG); a manually annotated and reviewed protein sequence (Swiss Prot) database; KEGG Ortholog database; and Gene Ontology (GO). GO enrichment analysis of the Differentially Expressed Genes (DEGs) was implemented using the GOseq R packages based on Wallenius non-central hyper-geometric distribution [61], which can adjust for gene length bias in DEGs. KEGG [62] is a database resource for understanding high-level functions and utilities of the biological system, such as the cell, organism, and ecosystem, from molecular-level information, especially large-scale molecular datasets generated by genome sequencing and other high-throughput experimental technologies (http://www.genome.jp/kegg/, accessed on 10 October 2022). We used KOBAS (https://academic.oup.com/bioinformatics/article/21/19/3787/210373) [63] software to test the statistical enrichment of DEGs in the KEGG pathways.

### 4.6. Phylogenetic Tree Construction, Sequence, and Structural Domain Alignment

To understand the homology of family genes and the conserved nature of the major structural domains, multiple sequence alignment was performed in this study using the full length of the PEPC protein sequence and the software DNAMAN (Version 7.0). The corresponding PEPC amino acid sequences were obtained from model crops, such as *Arabidopsis thaliana*, plus the *Dendrobium* PEPC protein. Multiple sequence alignment was performed using the default parameters of the ClusterW tool in MEGA 6.0, and the obtained results were subsequently used to generate a phylogenetic tree using the neighbor-joining method (bootstrap = 1, 000) [64]. The conserved PEPC protein sequences were identified and counted using the MEME online program (http://meme-suite.org/tools/meme, accessed on 22 June 2023) [65], with the conserved sequence length set to 6–50, the maximum number of identified sequences set to 10, and all other parameters set to default. The more conserved segments of the PEPC protein sequences were identified by MEME based on the parameter settings and distinguished by different colors.

### 4.7. MiRNA Target Site Analysis

MicroRNAs (miRNAs) play an important role in various pathways, including plant growth and development and metabolism. We downloaded the sequences of known mature miRNAs from the websites miRBase v 20.0 (http://www.mirbase.org, accessed on 17 May 2023) and PMRD (http://bioinformatics.cau.edu.cn/PMRD, accessed on 17 May 2023) for various crops. Using these known miRNAs to make predictions by association with different PEPC genes can identify which PEPC genes are the target genes for some of these miRNAs and which miRNAs may regulate the transcriptional translation of PEPC genes. This prediction was analyzed online via the psRNA Target website (https://www.zhaolab.org/psRNATarget, accessed on 23 June 2023) under the default parameters [66].

### 4.8. Real-Time PCR Analysis of PEPC

To identify DEGs, three genes of every species were selected, all related to the photosynthetic pathway. The Tiangen RNA Extraction Kit (DP441) (TIANGEN BIOTECH (BEIJING) Co., Ltd., Beijing, China) was employed to isolate total RNA from the samples. Following the manufacturer’s protocol, 1 μg of total RNA was used to synthesize cDNA using the Takara cDNA Synthesis Kit (Biological Engineering Company, Dalian, China), and fluorescent quantitative primers were designed using Primer 5.0. The designed primers are shown in Supplementary Table S1. For qRT-PCR, SYBR Green PCR reactions (Biological Engineering Company) and the ABI 7500 Fast Real Time PCR system were employed. The 20 μL reaction system contained Forward primer (0.4 μL), Reverse primer (0.4 μL), SYBR (10 μL), ROX (0.4 μL), H_2_O (6.8 μL), and cDNA (2 μL). The annealing temperature was set at 55 °C. Each sample was subjected to three replicates, and the relative expression level of each gene was calculated using the 2^−ΔΔCT^ method.

## 5. Conclusions

In summary, this study revealed 134.43 GB of sequencing data across the three *Dendrobium* species. Within this dataset, numerous DEGs exhibited distinct response mechanisms under drought stress. GO–KEGG-based enrichment analysis of these DEGs highlighted the involvement of metabolic pathways, including carbohydrate transport and metabolism, carbon metabolism, and photosynthesis, contributing to drought tolerance and alterations in photosynthetic pathways. Variations were observed in the structure and expression of PEPC genes under drought stress conditions among different *Dendrobium* species. Specifically, under these conditions, the PEPC genes, *Dc_gene2609* of *D. catenatum* and *c76953-c1* and *c78089-c1* of *D. loddigesii*, exhibited upregulated expression. Furthermore, the downregulation of dof-miR-384 expression led to the upregulation of *Dc_gene2609*, regulating the stomata closure and the conversion of the photosynthetic pathway from C_3_ to CAM. This adaptive response ultimately improved drought tolerance in *Dendrobium*.

## Figures and Tables

**Table 1:** Sample Information.

Sample	Species	Treatment
T1	Dendrobium fimbriatum Hook	WW
T2	Dendrobium fimbriatum Hook	WW
T3	Dendrobium fimbriatum Hook	WW
T4	Dendrobium fimbriatum Hook	DS
T5	Dendrobium fimbriatum Hook	DS
T6	Dendrobium fimbriatum Hook	DS
T07	Dendrobium loddigesii Rolfe	WW
T08	Dendrobium loddigesii Rolfe	WW
T09	Dendrobium loddigesii Rolfe	WW
T10	Dendrobium loddigesii Rolfe	DS
T11	Dendrobium loddigesii Rolfe	DS
T12	Dendrobium loddigesii Rolfe	DS
T13	Dendrobium catenatum Lindley	WW
T14	Dendrobium catenatum Lindley	WW
T15	Dendrobium catenatum Lindley	WW
T16	Dendrobium catenatum Lindley	DS
T17	Dendrobium catenatum Lindley	DS
T18	Dendrobium catenatum Lindley	DS

Note: WW and DS denotes Well-Watered and Drought-Stressed treatment, respectively.

**Table 2:** Statistics on sequencing data output of *Dendrobium catenatum*.

Sample	T13	T14	T15	T16	T17	T18
Clean Reads	25,736,675	24,698,887	23,658,162	25,059,306	23,723,106	22,974,697
Clean Bases	7,704,250,842	7,387,541,456	7,080,339,178	7,503,039,742	7,100,687,882	6,874,766,972
GC Content	45.57%	45.28%	45.82%	45.64%	46.07%	46.04%
≥Q30	92.66%	92.54%	92.75%	92.58%	92.34%	92.60%
Mapped Reads	84.81%	83.72%	84.90%	85.16%	85.31%	85.26%
Uniq Mapped Reads	82.13%	81.65%	82.21%	82.92%	82.69%	82.55%
Multiple Map Reads	2.68%	2.07%	2.69%	2.24%	2.62%	2.71%
Reads Map to “+”	42.09%	41.70%	42.15%	42.45%	42.45%	42.32%
Reads Map to “−”	42.18%	41.74%	42.25%	42.45%	42.51%	42.44%

Note: Samples: Sample name on the sample information sheet; Clean reads: The total number of pair end reads in Clean Data; Clean bases: Clean Data; GC content: GC content Clean Data, which is the percentage of G and C bases in Clean Data to the total base; ≥Q30: The percentage of bases with a Clean Data quality value of at least 30; Mapped Reads: The number of Reads mapped to the reference genome and their percentage in Clean Reads; Uniq Mapped Reads: The number of Reads already mapped to a unique position in the reference genome and their percentage in Clean Reads; Multiple Map Reads: The number of Reads that have been compared to multiple locations in the reference genome and their percentage in Clean Reads; Reads Map to “+”: the number of Reads aligned to the positive strand of the reference genome and their percentage in Clean Reads; Reads Map to “−”: The number of Reads aligned to the negative strand of the reference genome and their percentage in Clean Reads.

**Table 3:** Statistics on sequencing data output of *Dendrobium loddigesii* and *Dendrobium fimbriatum*.

Samples	Read Number	Base Number	GC Content	≥Q30
T1	25,222,133	7,549,261,904	45.97%	92.48%
T2	24,720,125	7,393,655,992	45.87%	92.03%
T3	24,779,891	7,403,415,432	45.63%	92.58%
T4	24,867,538	7,444,820,532	45.97%	92.49%
T5	26,247,245	7,850,055,066	45.85%	92.34%
T6	27,132,721	8,116,884,478	45.65%	92.27%
T07	25,019,702	7,491,393,560	45.56%	92.44%
T08	26,684,679	7,987,805,304	45.53%	92.48%
T09	24,734,656	7,404,300,212	45.25%	92.69%
T10	24,058,684	7,200,859,834	45.81%	92.61%
T11	23,864,004	7,139,039,344	45.14%	92.50%
T12	26,046,625	7,792,409,468	45.36%	92.40%

Note: Read Number: The total number of pair end reads in Clean Data; Base Number: Clean Data; GC Content: GC content Clean Data, which is the percentage of G and C bases in Clean Data to the total base; ≥Q30: The percentage of bases with a Clean Data quality value of at least 30.

## Data Availability

Data are contained within the article are available through the NCBI Sequence Read Archive under the accession number PRJNA976822.

## Supplementary Materials

The supporting information can be downloaded at: https://www.mdpi.com/article/10.3390/ijms25052731/s1.

## References

[1] Li Z., Liu C., Zhang Y., Wang B., Ran Q., Zhang J. (2019). The bHLH family member ZmPTF1 regulates drought tolerance in maize by promoting root development and abscisic acid synthesis. J. Exp. Bot..

[2] Zhang C., Shi S. (2018). Physiological and proteomic responses of contrasting alfalfa (*Medicago sativa* L.) varieties to PEG-induced osmotic stress. Front. Plant Sci..

[3] Opitz N., Marcon C., Paschold A., Malik W.A., Lithio A., Brandt R., Piepho H.P., Nettleton D., Hochholdinger F. (2016). Extensive tissue-specific transcriptomic plasticity in maize primary roots upon water deficit. J. Exp. Bot..

[4] Tripathi P., Rabara R.C., Shen Q.J., Rushton P.J. (2015). Transcriptomics analyses of soybean leaf and root samples during water-deficit. Genom. Data.

[5] Schuppler U., He P.H., John P.C.L., Munns R. (1998). Effect of water stress on cell division and cell-division-cycle 2-like cell-cycle kinase activity in wheat leaves. Plant Physiol..

[6] Gupta A., Rico-Medina A., Caño-Delgado A.I. (2020). The physiology of plant responses to drought. Science.

[7] Lu H.D., Xue J.Q., Guo D.W. (2017). Efficacy of planting date adjustment as a cultivation strategy to cope with drought stress and increase rainfed maize yield and water-use efficiency. Agric. Water Manag..

[8] Wu X., Yuan J., Luo A., Chen Y., Fan Y. (2016). Drought stress and re-watering increase secondary metabolites and enzyme activity in Dendrobium moniliforme. Ind. Crops Prod..

[9] Mahajan S., Tuteja N. (2005). Cold, salinity and drought stresses: An overview. Arch. Biochem. Biophys..

[10] Bota J., Medrano H., Flexas J. (2004). Is photosynthesis limited by decreased RuBisCO activity and RuBP content under progressive water stress?. New Phytol..

[11] Hoekstra F.A., Golovina E.A., Buitink J. (2001). Mechanisms of plant desiccation tolerance. Trends Plant Sci..

[12] Chaves M.M., Maroco J.P., Pereira J.S. (2003). Understanding plant responses to drought—From genes to the whole plant. Funct. Plant Biol..

[13] Zhu J.K. (2016). Abiotic stress signaling and responses in plants. Cell.

[14] Khanna-Chopra R., Singh K., Tripathi B.N., Müller M. (2015). Drought Resistance in Crops: Physiological and Genetic Basis of Traits for Crop Productivity Stress Responses in Plants.

[15] Qiu S., Xia K., Yang Y., Wu Q., Zhao Z. (2023). Mechanisms underlying the C_3_–CAM photosynthetic shift in facultative CAM plants. Horticulturae.

[16] He J., Chua E.L., Qin L. (2020). Drought does not induce crassulacean acid metabolism (CAM) but regulates photosynthesis and enhances nutritional quality of Mesembryanthemum crystallinum. PLoS ONE.

[17] Ahmad P. (2016). Water Stress and Crop Plants: A Sustainable Approach.

[18] Huang H., Wang H., Tong Y., Wang Y. (2020). Insights into the superoxide dismutase gene family and its roles in Dendrobium catenatum under abiotic stresses. Plants.

[19] Lim G.H., Kim S.W., Ryu J., Kang S.Y., Kim J.B., Kim S.H. (2020). Upregulation of the MYB2 transcription factor Is associated with increased accumulation of anthocyanin in the leaves of Dendrobium bigibbum. Int. J. Mol. Sci..

[20] Gong X., Jiang S., Tian H., Xiang D., Zhang J. (2020). Polyphenols in the fermentation liquid of Dendrobium candidum relieve intestinal inflammation in zebrafish through the intestinal microbiome-mediated immune response. Front. Immunol..

[21] Yang M.Z., Shan Y.Y., Chen X.M., Zhang C.F., Li Z.J. (2022). Current development situation of dendrobium industry in China. Mod. Chin. Med..

[22] Wang S.S., Liu J.M., Sun J., Sun Y.F., Liu J.N., Jia N., Fan B., Dai X.F. (2019). Diversity of culture-independent bacteria and antimicrobial activity of culturable endophytic bacteria isolated from different Dendrobium stems. Sci. Rep..

[23] Zhang Y., Li Y.Y., Chen X.M., Guo S.X., Lee Y.I. (2020). Effect of different mycobionts on symbiotic germination and seedling growth of Dendrobium officinale, an important medicinal orchid. Bot. Stud..

[24] Yeow L.C., Chew B.L., Sreeramanan S. (2020). Elevation of secondary metabolites production through light-emitting diodes (LEDs) illumination in protocorm-like bodies (PLBs) of Dendrobium hybrid orchid rich in phytochemicals with therapeutic effects. Biotechnol. Rep..

[25] Ng C.K.Y., Hew C.S. (2000). Orchid pseudobulbs—‘False’ bulbs with a genuine importance in orchid growth and survival!. Sci. Hortic..

[26] Zimmerman J.K. (1990). Role of pseudobulbs in growth and flowering of *Catasetum viridiflavum* (Orchidaceae). Am. J. Bot..

[27] Zhang S.B., Dai Y., Hao G.Y., Li J.W., Fu X.W., Zhang J.L. (2015). Differentiation of water-related traits in terrestrial and epiphytic Cymbidium species. Front. Plant Sci..

[28] Cai J., Liu X., Vanneste K., Proost S., Tsai W.C., Liu K.W., Chen L.J., He Y., Xu Q., Bian C. (2015). The genome sequence of the orchid Phalaenopsis equestris. Nat. Genet..

[29] Zhang G.Q., Xu Q., Bian C., Tsai W.C., Yeh C.M., Liu K.W., Yoshida K., Zhang L.S., Chang S.B., Chen F. (2016). The Dendrobium catenatum Lindl. Genome sequence provides insights into polysaccharide synthase, floral development and adaptive evolution. Sci. Rep..

[30] Zhang Z., He D., Niu G.H., Gao R.F. (2014). Concomitant CAM and C_3_ photosynthetic pathways in Dendrobium officinale Plants. J. Am. Soc. Hortic. Sci..

[31] Qiu S., Sultana S., Liu Z.D., Yin L.Y., Wang C.Y. (2015). Identification of obligate C_3_ photosynthesis in Dendrobium. Photosynthetica.

[32] Davis S.C., Simpson J., Gil-Vega K.D.C., Niechayev N.A., Tongerlo E.V., Castano N.H., Dever L.V., Búrquez A. (2019). Undervalued potential of crassulacean acid metabolism for current and future agricultural production. J. Exp. Bot..

[33] Bayramov S. (2017). Changes in protein quantities of phosphoenolpyruvate carboxylase and RuBisCO Activase in various wheat genotypes. Saudi J. Biol. Sci..

[34] Ping C.Y., Chen F.C., Cheng T.C., Lin H.L., Lin T.S., Yang W.J., Lee Y.I. (2018). Expression profiles of phosphoenolpyruvate carboxylase and phosphoenolpyruvate carboxylase kinase genes in Phalaenopsis, implications for regulating the performance of crassulacean acid metabolism. Front. Plant Sci..

[35] Punchkhon C., Plaimas K., Buaboocha T., Siangliw J.L., Toojinda T., Comai L., De Diego N., Spíchal L., Chadchawan S., Gene D.-T. (2020). Drought-tolerance gene identification using genome comparison and co-expression network analysis of chromosome substitution lines in rice. Genes.

[36] Yan L., Wang X., Liu H., Tian Y.L., Lian J.M., Yang R.J., Hao S.M., Wang X., Yang S., Li Q. (2015). The genome of Dendrobium officinale illuminates the biology of the important traditional Chinese orchid herb. Mol. Plant.

[37] Zhang G.Q., Liu K.W., Li Z., Lohaus R., Hsiao Y.Y., Niu S.C., Wang J.Y., Lin Y.C., Xu Q., Chen L.J. (2017). The Apostasia genome and the evolution of orchids. Nature.

[38] Zhang J.X., He C.M., Wu K.L., Teixeira da Silva J.A., Zeng S.J., Zhang X.H., Yu Z.M., Xia H.Q., Duan J. (2016). Transcriptome analysis of Dendrobium officinale and its application to the identification of genes associated with polysaccharide synthesis. Front. Plant Sci..

[39] Winter K., Wallace B.J., Stocker G.C., Roksandic Z. (1983). Crassulacean acid metabolism in Australian vascular epiphytes and somerelated species. Oecologia.

[40] Silvera K., Santiago L.S., Winter K. (2005). Distribution ofcrassulacean acid metabolism in orchids of Panama:evidence of selection of weak and strong modes. Funct. Plant Biol..

[41] Yuan G., Hassan M.M., Liu D., Lim S.D., Yim W.C., Cushman J.C., Markel K., Shih P.M., Lu H., Weston D.J. (2020). Biosystems design to accelerate C_3_-to-CAM progression. BioDesign Res..

[42] Li Q., Liu C., Huang C., Wang M., Long T., Liu J., Shi J., Shi J., Li L., He Y. (2022). Transcriptome and metabonomics analysis revealed the molecular mechanism of differential metabolite production of Dendrobium nobile under different epiphytic patterns. Front. Plant Sci..

[43] Yuan Y., Zuo J., Zhang H., Li R., Yu M., Liu S. (2022). Integration of transcriptome and metabolome provides new insights to flavonoids biosynthesis in Dendrobium huoshanense. Front. Plant Sci..

[44] Wan X., Zou L.H., Zheng B.Q., Tian Y.Q., Wang Y. (2018). Transcriptomic profiling for prolonged drought in Dendrobium catenatum. Sci. Data.

[45] Rabas A.R., Martin C.E. (2003). Movement of water from old to young leaves in three species of succulents. Ann. Bot..

[46] Borland A.M., Taybi T. (2004). Synchronization of metabolic processes in plants with crassulacean acid metabolism. J. Exp. Bot..

[47] Arias-Moreno D.M., Jiménez-Bremont J.F., Maruri-López I., Delgado-Sánchez P. (2017). Effects of catalase on chloroplast arrangement in Opuntia streptacantha chlorenchyma cells under salt stress. Sci. Rep..

[48] Chi Z., Wang Z.P., Wang G.Y., Khan I., Chi Z.M. (2016). Microbial biosynthesis and secretion of l-malic acid and its applications. Crit. Rev. Biotechnol..

[49] Müller G.L., Lara M.V., Oitaven P., Andreo C.S., Maurino V.G., Drincovich M.F. (2018). Improved water use efficiency and shorter life cycle of *Nicotiana tabacum* due to modification of guard and vascular companion cells. Sci. Rep..

[50] Stevens G.G., Pérez-Fernández M.A., Morcillo R.J.L., Kleinert A., Hills P., Brand D.J., Steenkamp E.T., Valentine A.J. (2019). Roots and nodules response differently to P starvation in the Mediterranean-type legume Virgilia divaricata. Front. Plant Sci..

[51] Izui K., Matsumura H., Furumoto T., Kai Y. (2004). Phosphoenolpyruvate carboxylase: A new era of structural biology. Annu. Rev. Plant Biol..

[52] Doubnerová Hýsková V., Miedzińska L., Dobrá J., Vankova R., Ryšlavá H. (2014). Phosphoenolpyruvate carboxylase, NADP-malic enzyme, and pyruvate, phosphate dikinase are involved in the acclimation of *Nicotiana tabacum* L. to drought stress. J. Plant Physiol..

[53] Cheng G., Wang L., Lan H. (2016). Cloning of PEPC-1 from a C4 halophyte Suaeda aralocaspica without Kranz anatomy and its recombinant enzymatic activity in responses to abiotic stresses. Enzyme Microb. Technol..

[54] Helwak A., Kudla G., Dudnakova T., Tollervey D. (2013). Mapping the human miRNA interactome by CLASH reveals frequent noncanonical binding. Cell.

[55] Li Y., Wang X., Guo Q., Zhang X., Zhou L., Zhang Y., Zhang C. (2022). Conservation and diversity of miR166 family members from highbush blueberry (*Vaccinium corymbosum*) and their potential functions in abiotic stress. Front. Genet..

[56] Vaasen A., Begerow D., Hampp R. (2006). Phosphoenolpyruvate carboxylase genes in C_3_, crassulacean acid metabolism(CAM) and C_3_/CAM intermediate species of the genus Clusia: Rapid reversible C_3_/CAM switches are based on the C_3_ housekeeping gene. Plant Cell Environ..

[57] Gehrig H., Faist K., Kluge M. (1998). Identification of phosphoenolpyruvate carboxylase isoforms in leaf, stemand roots of the obligate CAM plant Vanilla planifolia Salib.(Orchidaceae): A physiological and molecular approach. Plant Mol. Biol..

[58] Hoagland D.R., Snyder W.C. (1933). Nutrition of strawberry plant under controlled conditions. (a) Effects of deficiencies of boron and certain other elements, (b) Susceptibility to injury from sodium salts. Proc. Am. Soc. Hortic. Sci..

[59] Grabherr M.G., Haas B.J., Yassour M., Levin J.Z., Thompson D.A., Amit I., Adiconis X., Fan L., Raychowdhury R., Zeng Q.D. (2011). Full-length transcriptome assembly from RNA Seq data without a reference genome. Nat. Biotechnol..

[60] Anders S., Pyl P.T., Huber W. (2015). HTSeq—A Python framework to work with high-throughput sequencing data. Bioinformatics.

[61] Young M.D., Wakefield M.J., Smyth G.K., Oshlack A. (2010). Gene ontology analysis for RNA-seq: Accounting for selection bias. Genome Biol..

[62] Kanehisa M., Araki M., Goto S., Hattori M., Hirakawa M., Itoh M., Katayama T., Kawashima S., Okuda S., Tokimatsu T. (2008). KEGG for linking genomes to life and the environment. Nucleic Acids Res..

[63] Mao X., Cai T., Olyarchuk J.G., Wei L. (2005). Automated genome annotation and pathway identification using the KEGG orthology (KO) as a controlled vocabulary. Bioinformatics.

[64] Tamura K., Stecher G., Peterson D., Filipski A., Kumar S. (2013). MEGA6: Molecular evolutionary genetics analysis version 6.0. Mol. Biol. Evol..

[65] Bailey T.L., Elkan C. (1994). Fitting a mixture model by expectation maximization to discover motifs in biopolymers. Proc. Int. Conf. Intell. Syst. Mol. Biol..

[66] Zhang Y. (2005). miRU: An automated plant miRNA target prediction server. Nucleic Acids Res..

