# Supplementary figures and images for: Drought Stress Induced Different Response Mechanisms in Three Dendrobium Species under Different Photosynthetic Pathways
Source: Int J Mol Sci. 2024 Feb 27;25(5):2731. doi: 10.3390/ijms25052731 (PMC10932418; doi:10.3390/ijms25052731)

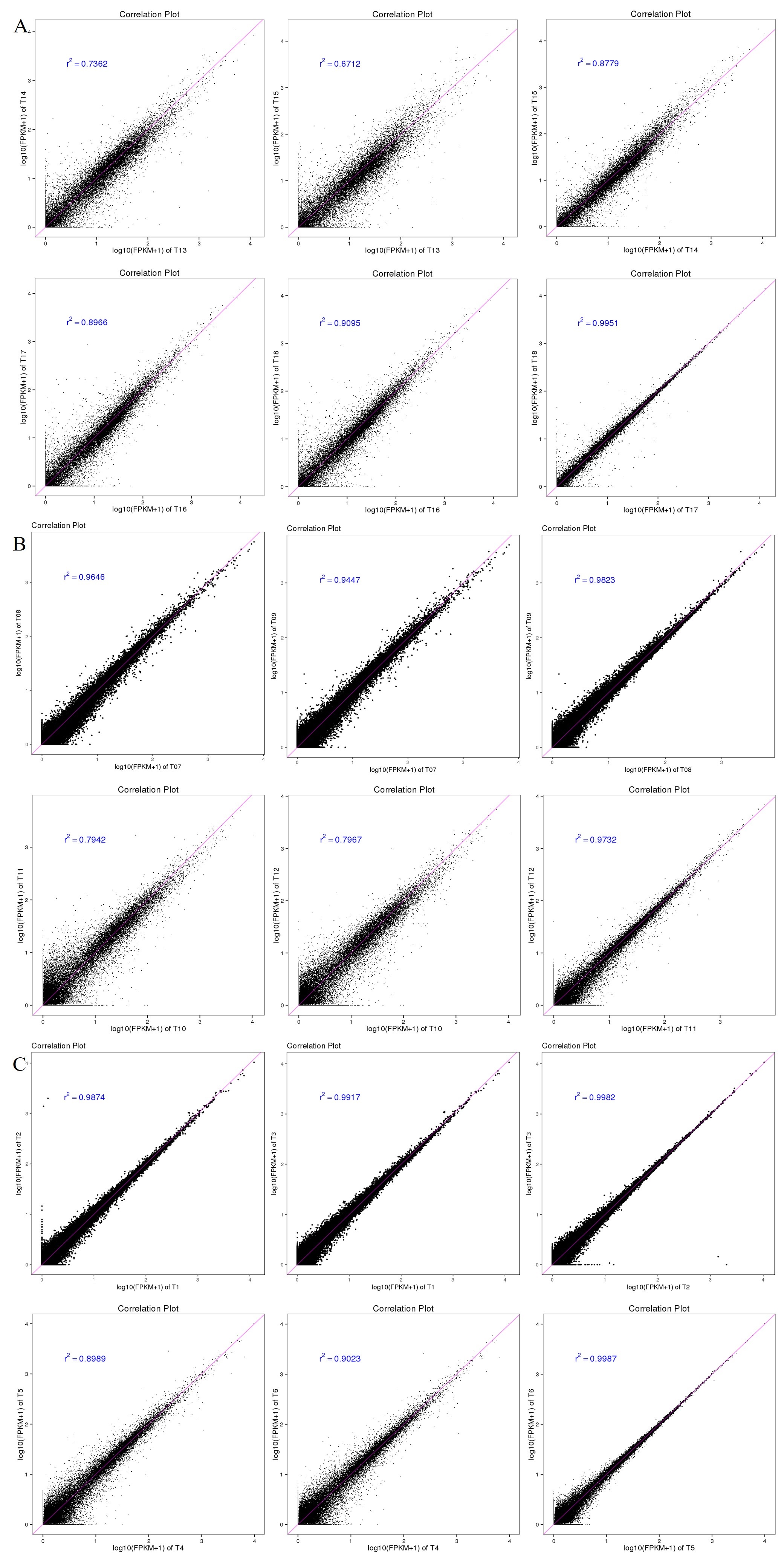

Supplement: Supplementary file 1 [file ijms-25-02731-s001.zip › Supplemental Figure S1.jpg]

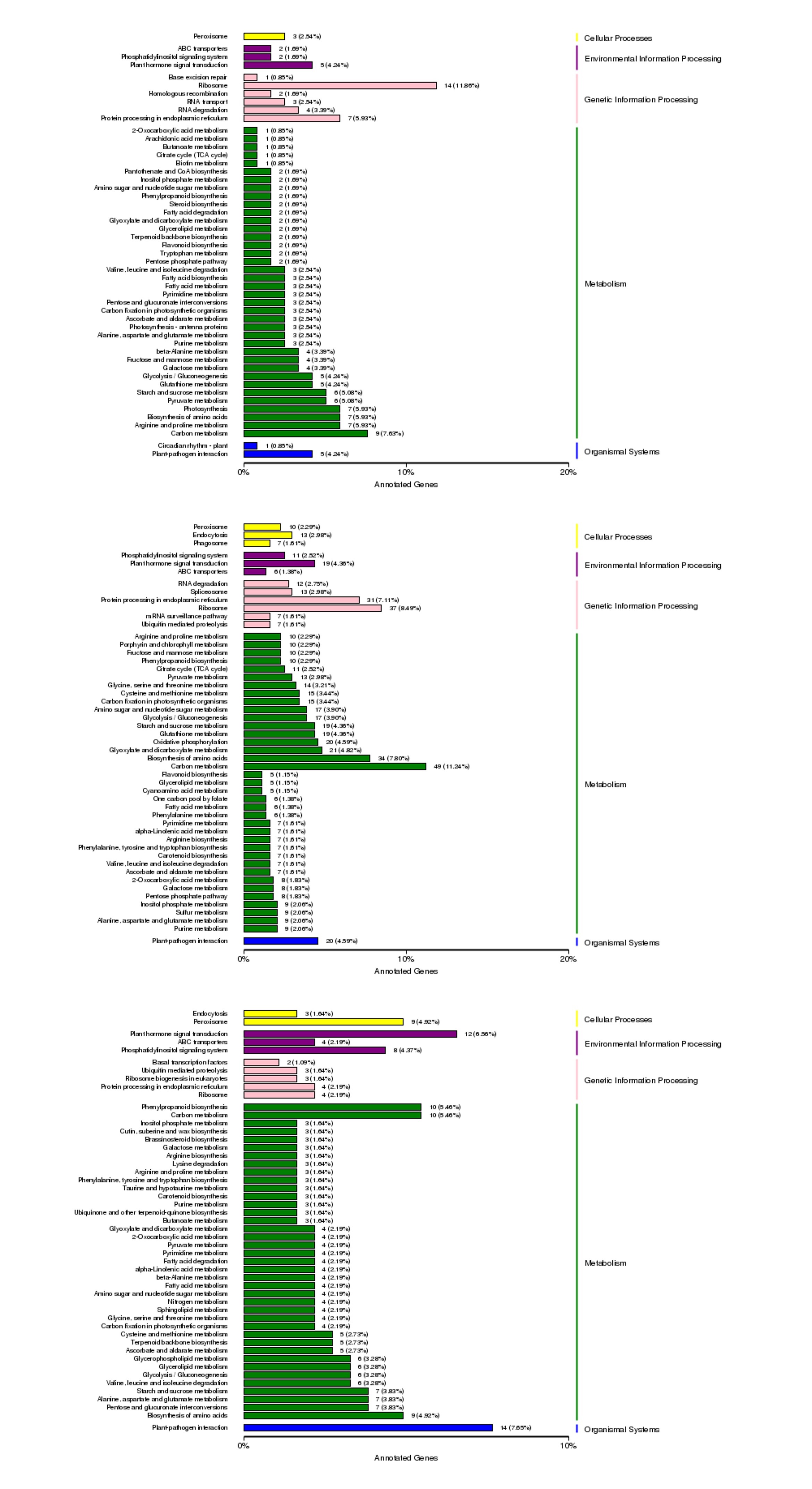

Supplement: Supplementary file 1 [file ijms-25-02731-s001.zip › Supplemental Figure S2.jpg]

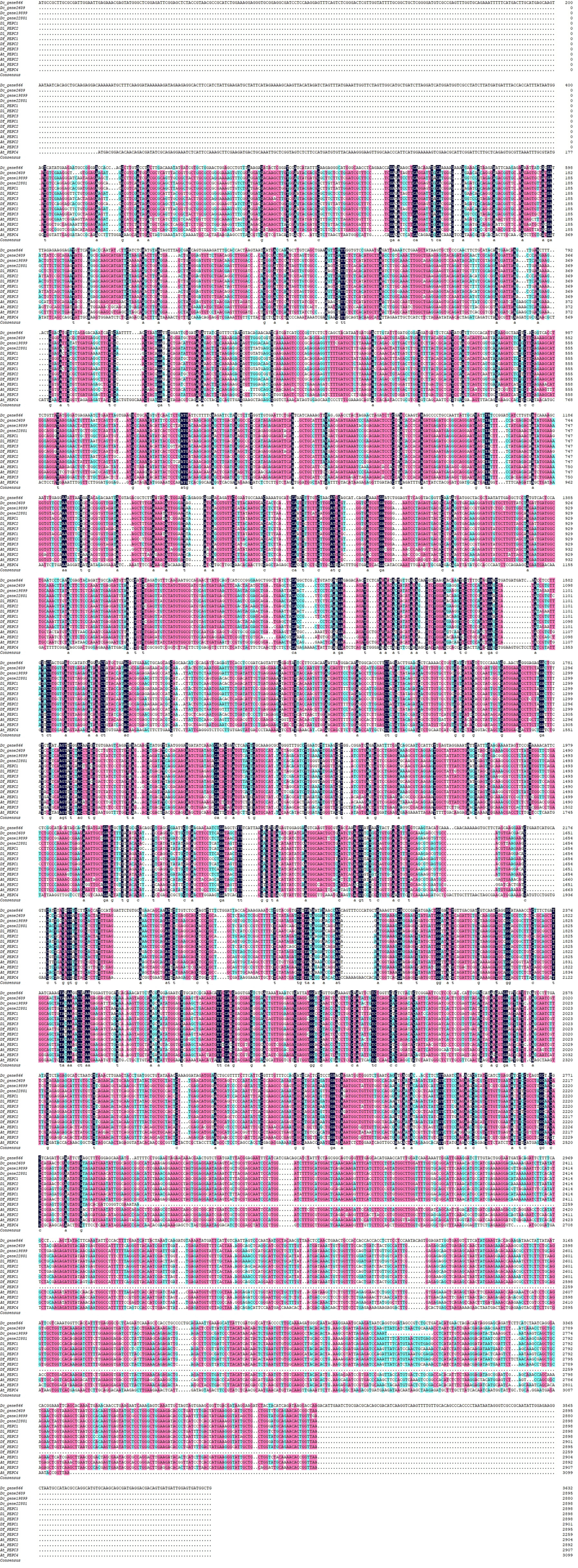

Supplement: Supplementary file 1 [file ijms-25-02731-s001.zip › Supplemental Figure S4.jpg]

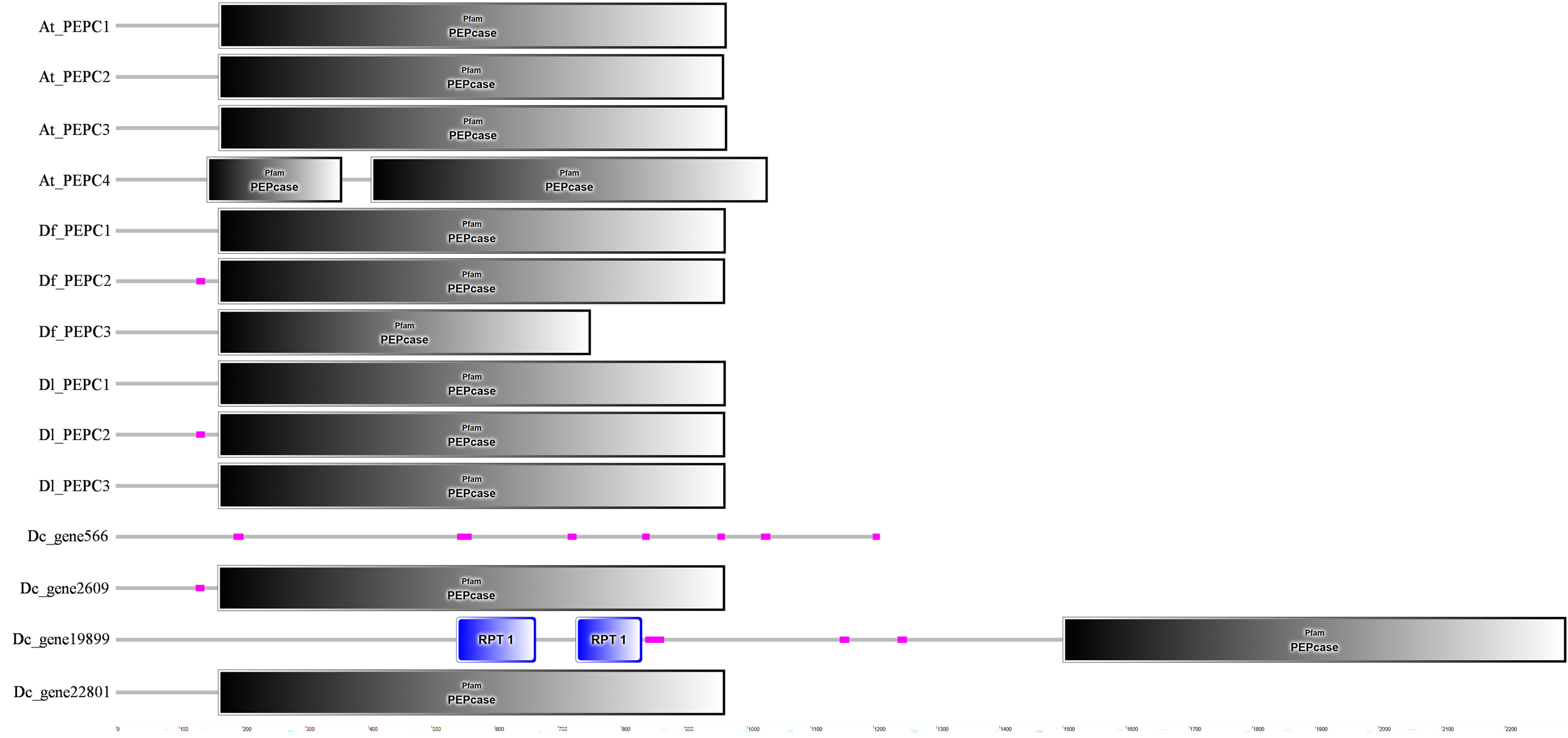

Supplement: Supplementary file 1 [file ijms-25-02731-s001.zip › Supplemental Figure S5.jpg]
